# Real-Life Experience in the Management of Sinonasal Complications of Dental Disease or Treatments

**DOI:** 10.3390/jpm12122078

**Published:** 2022-12-16

**Authors:** Eugenio De Corso, Mario Rigante, Dario Antonio Mele, Stefano Settimi, Daniele Penazzi, Carlo Lajolo, Massimo Cordaro, Marco Panfili, Claudio Montuori, Jacopo Galli, Gaetano Paludetti

**Affiliations:** 1 Unit of Otorhinolaryngology, Fondazione Policlinico Universitario A.Gemelli IRCCS, 00168 Rome, Italy; 2 Head and Neck and Sensory Organs Department, Università Cattolica del Sacro Cuore, 00168 Rome, Italy; 3 Head and Neck Department, Fondazione Policlinico Universitario A. Gemelli IRCCS, School of Dentistry, Università Cattolica del Sacro Cuore, 00168 Rome, Italy; 4 Unit of Radiology, “A. Gemelli” University Hospital Foundation IRCCS, 00168 Rome, Italy

**Keywords:** maxillary odontogenic sinusitis, oroantral fistula, maxillary sinusitis, dental implants, SNOT-22

## Abstract

Diagnosis and management of sinonasal complications of dental diseases or treatment (SCDDT) may be challenging. We aimed to report our real-life experience in patients treated with endoscopic endonasal approach describing data about symptoms, etiology, extension of the disease and success rate. We evaluated retrospectively data about 262 patients diagnosed as SCDDT and managed from August 2015 to May 2022. In 44.65% cases, maxillary sinus complications were determined by a dental disorder; the remaining 55.34% of cases were iatrogenic. Patients were managed according to our multidisciplinary protocol including ENT, dental, and radiological evaluation. Treatments were planned with a personalized approach, based on the patient’s clinical characteristics; all patients were treated with an endonasal endoscopic mini-invasive conservative approach. Combined dental treatment was performed simultaneously in 152/262 (58%) of patients; in the remaining cases, it was postponed after surgery. The overall treatment success rate (symptom resolution and endoscopically observed maxillary sinus healing) was 96.5%. At 15 days after surgery, we observed a significant improvement in the quality of life. The mean post-operative Sinonasal outcome test-22 (SNOT-22) score was significantly lower compared to baseline (6 versus 43.4; *p* < 0.05). Our study showed that endoscopic sinus surgery can be a successful procedure for treatment of SCDDT, leading to fast resolution of sinonasal symptoms and improving the quality of life. Furthermore, the technique allows removal of migrated dental material or dental implants even in challenging cases.

## 1. Introduction

Sinusitis of dental origin is a well-recognized condition in both dental, maxillo-facial, and ENT communities. It accounts for 10% of all cases of maxillary sinusitis in Europe and for 14% in USA [1]. It is also known as odontogenic maxillary sinusitis (OMS). Recently, Felisati et al. [2] tried to integrate the definition by adopting the acryonym SCDDT (sinonasal complications of dental disease or treatment) in order to include implant and sinus augmentation-related etiologies [2,3,4]. This new classification [2] is based on the assumption that any disease or treatment of dental or dento-alveolar structures close to the maxillary sinus may affect the integrity of the Schneiderian membrane (SM), leading to different maxillary complications. The interruption of the mucoperiosteum is generally associated with a high probability of infections, especially by anaerobes bacteria or unusual oral pathogens [5,6].

Several etiologies of SCDDT have been proposed [7]. First of all, maxillary complications may depend on inflammatory dental and periodontal pathologies such as dental caries, endodontic infection with pulp and periapical complications (granulomas or small inflammatory cysts), complex endo-parodontal lesions (periodontal pocket), and large odontogenic cysts [8,9]. In addition, there is an increase in “iatrogenic causes” including dental implants (dimensions and axis not adapted/late migration into the maxillary sinus), pre-implantologic maxillary sinus lift, foreign bodies (dental fillings, tooth roots, parts of broken instruments) pushed through the root canal or by oroantral fistula [10]. Finally, oroantral fistula should be also considered due to the large number of extractions of posterior teeth. The iatrogenic damage may be related to the thinness of the antral floor in that region ranging from 1 to 7 mm. Even if the incidence is relatively low (5%), it may predispose to chronic maxillary rhinosinusitis [11].

While SCDDT is not uncommon and multiple studies have shown that it is one of the most frequent causes of unilateral maxillary sinus opacification in the adult population, its diagnosis can be elusive due to nonspecific sinonasal symptoms and minimal dental complaints [12,13]. The classic suggestive presentation includes both sinonasal symptoms (nasal obstruction, rhinorrhea, and/or foul odor and taste) and dental symptoms (pain and dental hypersensitivity), even though the latter do not reliably predict an odontogenic cause [5,14]. Allevi et al. [15], in a systematic review of the literature, observed extreme heterogeneity in diagnostic criteria and emphasized the necessity of consensus that defines both sinusitis and related odontogenic foci. Felisati et al. in 2013 [2] focused attention on importance of multidisciplinary treatment, allowing a combination of different surgical skills in a single procedure and reduction of rehabilitation times.

Several surgical approaches have been proposed over the years for treatment of SCDDT. External approach and extensive exploration of the affected sinus were widely employed in the past, although these methods are traumatic and associated with important postoperative complications, such as trigeminal neuralgia [16,17]. In more recent years, endoscopic endonasal approaches have become widely diffused, thanks to minimal invasiveness, less morbidity, and a lower incidence of complications [18,19]. In addition, cooperation between ENT and dentists can help to guarantee the best outcomes.

The objective of this study was to describe our real-life experience in the management of SCDDT treated with an endoscopic endonasal approach at our institution, describing epidemiological data about symptoms, etiology, extension and success rate.

## 2. Materials and Methods

This was a monocentric retrospective study including patients affected by SCDDT and managed in a real-life setting according to the type of disease at our tertiary academic center (Department of Otorhinolaryngology—Head and Neck Surgery—A. Gemelli University Hospital Foundation IRCCS) from August 2015 to May 2022.

We included all patients who underwent endoscopic sinus surgery for the treatment of SCDDT at our institution. The diagnosis was confirmed in all patients by CT, nasal endoscopy, and dental evaluation. The clinical charts of ENT patients were retrospectively gathered. Most patients were initially diagnosed at our department (75.5%), while the remaining (24.5%) were referred to our attention from a dentist’s clinic. The study was approved by our Institutional Review Board and written informed consent was obtained from all patients.

At our institution, patients with suspected SCDDT are usually managed according to a multidisciplinary approach. ENT evaluation at baseline includes a general health assessment and specific rhinologic analysis of signs and symptoms related to paranasal sinus infection, such as mucopurulent rhinorrhea, and chronic or intermittent facial pain. ENT clinical evaluation is always associated with nasal endoscopy (Karl Storz GmbH & Co. KG, Tuttlingen, Germany) that may help in diagnosis by showing unilateral purulent rhinorrhea or meatal edema/polyps. We usually evaluate the subjective burden of patients’ symptoms using the validated SNOT-22 questionnaire that is specific for burden of sinonasal symptoms on quality of life [20]. It is a questionnaire composed by 22 Chronic Rhinosinusitis(CRS)-related items scored from 0 to 5 (total score range 0–110, higher scores represent worse symptoms). Patients answer to the items (under investigators supervision) pre-operatively and 15 days after the procedure, in order to evaluate the effects on the Quality of Life after a short period from surgery.

The baseline evaluation by a dentist is focused on odontogenic disease that gave rise to the maxillary complication and analyzes the oral and parodontal status as well as the presence of oroantral communications: after a careful medical history, a thorough clinical examination was carried out by a well trained dentist with at least 5 years of clinical practice in order to detect tooth pathologies connected to the sinus disease: especially, the presence of deep decays, cracked teeth, oro-antral fistulae, inflammatory fistulae were checked especially in the side of the sinus pathology; thermal tests, percussion tests, and periodontal probing were performed in the upper teeth; Valsalva maneuver was also performed to detect small oro-antral fistulae. Furthermore, specific radiologic assessment with panorex, conventional CT or cone beam computed tomography (CBCT) is performed by a dedicated head and neck radiologist. We always perform radiologic exams as an essential support to diagnostic work-up for evaluating the extent of the disease (considering axial, sagittal and coronal planes), and they are useful to guide subsequent treatment. During planning of SCDDT management, patients are always involved in the decision-making protocol, sharing the timing and modality of dental/ENT treatments. We usually prefer an endoscopic mini-invasive endonasal approach to treat the sinuses involved. A combined ENT/odontoiatric approach in the same surgery is usually planned when possible, otherwise specific odontoiatric treatment is postponed after surgery based on the specific needs of patients. Timing of multidisciplinary interventions is scheduled by trying to tailor treatments considering the patient’s characteristics and needs in the view of personalized treatment. In case of complications after dental implant placement, the multidisciplinary team usually tries to preserve the dental implant, because its removal may be associated with an increased risk of oroantral fistula (OAF), more challenging reimplantation, and significant costs for the patient. In case of dislocation of the implant into the sinus, the possibility of endoscopic removal is always taken into account.

All patients signed informed consent forms before treatments and all received pre- and post-surgical oral antibiotic therapy for 5 and 10 days, respectively. Endoscopic sinus surgery was performed in all cases under general anesthesia with oro-tracheal intubation. First, inferior uncinectomy was performed to expose the ostium; polypoid tissue was removed if present and a wide middle antrostomy was performed in order to facilitate removal of pus, infected grafting material, and foreign bodies that had migrated inside the maxillary sinus. No attempt to entirely remove the sinus mucosa (different from the traditional Caldwell–Luc technique) was carried out. In cases of extra-maxillary sinus involvement, all the affected sinuses were treated. Removed tissue was always sent for histopathological examination. In order to eliminate every possible obstacle to functional recovery of the involved sinus, all concomitant conditions (septal deviation, concha bullosa, middle turbinate malformations or hypertrophy) were treated.

In order to evaluate the success rate, we considered as the following as the main outcomes: resolution of symptoms of sinusitis (anterior and posterior drainage, nasal obstruction, facial pressure, smell loss, and foul smell), significant improvement of quality of life measured by the SNOT-22 at 15 days after surgery (more that 8.9 points-minimally significant change of the score), and resolution of endoscopic findings in terms of purulent exudate, oedema, and polyps.

The statistical analysis was performed using SPSS for Windows (IBM Corp, Chicago, IL, USA). Normality of SNOT-22 results was verified with the Shapiro-Wilk test (normal for *p* > 0.05). T-test for paired samples was used for normally distributed data. All results are reported as mean ± standard deviation (SD). Statistical significance was assumed for *p*-values < 0.05.

## 3. Results

We enrolled 262 patients (female: 156, male: 106; mean age: 50.84 years) with a diagnosis of sinonasal complications of dental disease or treatment and treated by an endoscopic endonasal approach. All patients had unilateral symptoms. Analyzing the predominant symptoms, we observed nasal obstruction in 161/262 (61.4%) of patients, and anterior and posterior discharge in 226/262 cases (86.3%) and 196/262 cases (74.8%), respectively; 168/262 (65.2%) patients complained about smell disorders (43.4% cacosmia and only 21.6% hyposmia). Facial pressure or pain was registered in 133/262 (50.8%) of cases. The assessment of quality of life was performed using the validate SNOT-22 questionnaire and we observed a mean preoperative SNOT-22 of 43.4. Endoscopic examination showed the presence of a hyperplastic mucosa with edema in 129/262 (49.2%) cases, while polyps were present only in 48/262 (18.3%) cases. Purulent exudate was the most frequent finding observed in 224/262 patients (85.5%). The extension of the disease was evaluated by CT and in terms of involvement of paranasal sinuses we observed the following: maxillary 101/262 (38.5%); maxillary + ethmoid 132/262 (50.4%); maxillary + ethmoid + sphenoid 3/262 (1.1%); maxillary + ethmoid + frontal 26/262 (9.9%) (Table 1).

According to Felisati et al. [2], we classified patients based on the type of complication. Most patients (67.9%) had classic dental treatment complications (Group III), with (Class 3a) or without (Class 3b) oroantral communication. Pre-implantological treatment complications (Group I) were observed in 8.4% of cases and implantological complications in a total of 23.7% (Group II). The number of patients of additional sub-classes is reported in Table 2.

**Figure 1:**
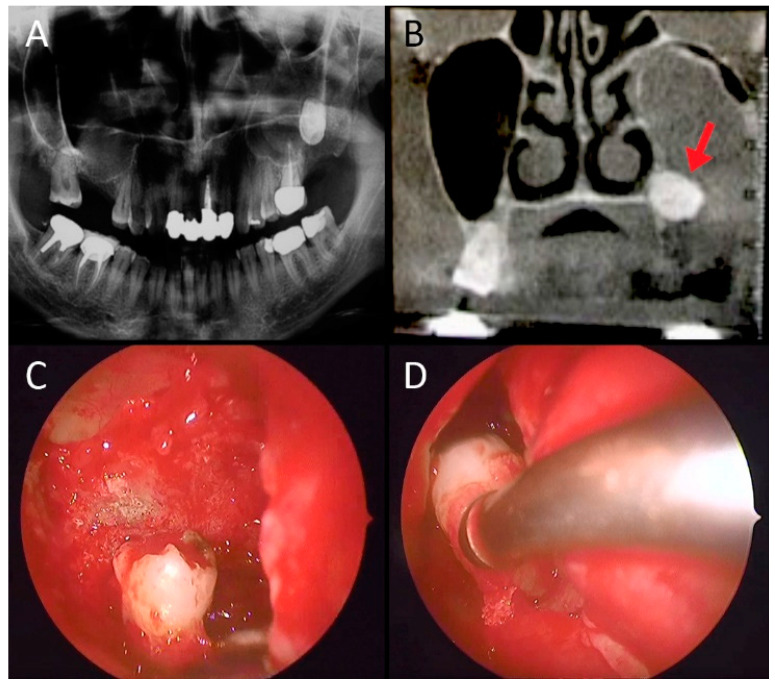
Male, 46 years old, complaining of nasal obstruction, rhinorrhea and foul odor and taste, without dental symptoms. The panoramic X-ray (**A**) and the CT scan (**B**) revealed a large odontogenic cyst of the left maxillary sinus, with an impacted tooth inside (red arrow in (**B**)). An exclusive endoscopic endonasal surgical approach was chosen; the tooth (**C**) was therefore removed (**D**) together with the cyst endoscopically.

**Figure 2:**
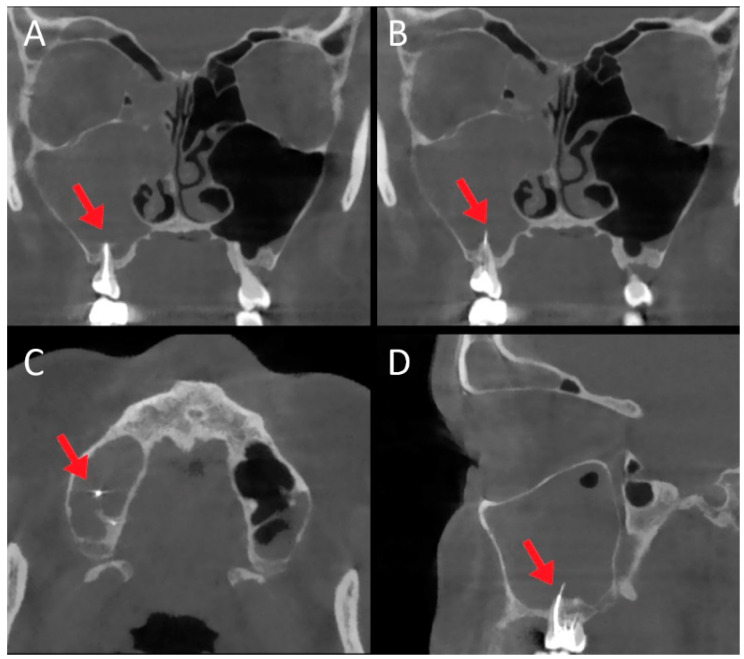
Female patient, 52 years old, complaining of facial pain, nasal obstruction, and rhinorrhea with a history of endodontic treatment 5 months earlier. CT ((**A**,**B**): coronal scan; (**C**): axial scan; (**D**): sagittal scan) revealed chronic right maxillary sinusitis extending to the right frontal sinus, and the presence of endodontic material in the right maxillary sinus (red arrows). Endoscopic surgery was performed before subsequent revision of dental surgery.

From an etiological point of view, in 117/262 (44.65%) cases the cause of maxillary sinusitis was dental disorder such as dentigenous cyst or tooth intrusion in 35/117 (29.9%) patients, radicular cyst in 29/117 cases (24.8%), dental caries in 12/117 cases (10.2%), and complication of endodontic treatment in 41/117 (35%) patients. In these patients, the distribution of involved teeth was as follows: the 2nd molar in 48/117 cases (41%), the 1st molar in 39/117 (33.3%), the 2nd premolar and 1st molar in 13/117 (11.1%), the 1st molar and 2nd molar in 9/117 (7.7%), the 2nd premolar in 4/117 (3.4%), and the 3rd molar in 4/117 cases (3.4%). In these patients, therapeutic modalities included trans-nasal endoscopic sinus surgery and dental management. Figure 1 and Figure 2 show a case of large odontogenic cyst of the left maxillary sinus and a a complication of endodontic treatment, respectively.

**Figure 3:**
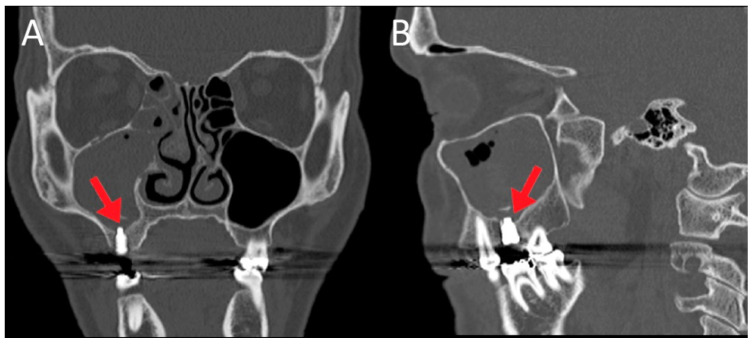
Female patient, 64 years old, who had undergone dental implant surgery 3 months earlier, complaining of left facial pain. CT (**A**): coronal scan; (**B**): sagittal scan) revealed odontogenic right maxillary sinusitis (red arrows indicate the dental implant). No migration of the dental implant was documented. An endoscopic primary surgical approach was proposed. The removal of implant removal was not necessary.

**Figure 4:**
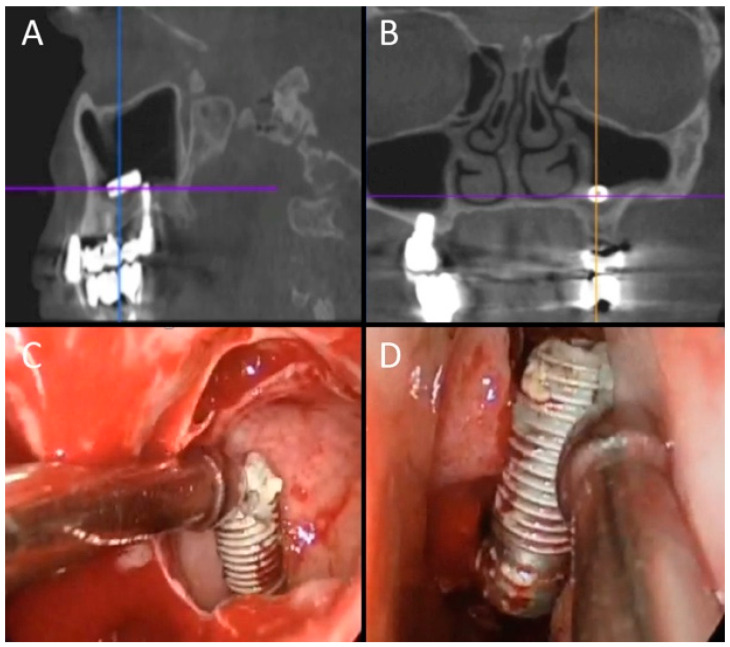
Female patient, 58 years old, who had undergone dental implant surgery several years earlier. CT performed for sinus pain documented migration into the left maxillary sinus ((**A**): sagittal scan; (**B**): coronal scan). An endoscopic surgical approach achieved removal of the foreign body ((**C**) and (**D**)).

In 145 of 262 (55.34%) patients, the cause of maxillary sinusitis was iatrogenic. Dental implant-related complications were found in 62/145 patients (42.7%), dental extraction-related complications were found in 50/145 cases (34.5%), and foreign bodies in 19/145 cases (13.1%). The distribution of involved teeth in this group was as follows: the 2nd molar in 59/145 (40.7%), the 1st molar in 48/145 (33.1%), the 2nd premolar and 1st molar in 16 (11.1%), the 1st molar and 2nd molar in 10/145 (6.9%), the 2nd premolar in 6/145 (4.1%), and the 3rd molar in 6/145 cases (4.1%). Figure 3 and Figure 4 show two cases of complications after implant placement.

**Figure 5:**
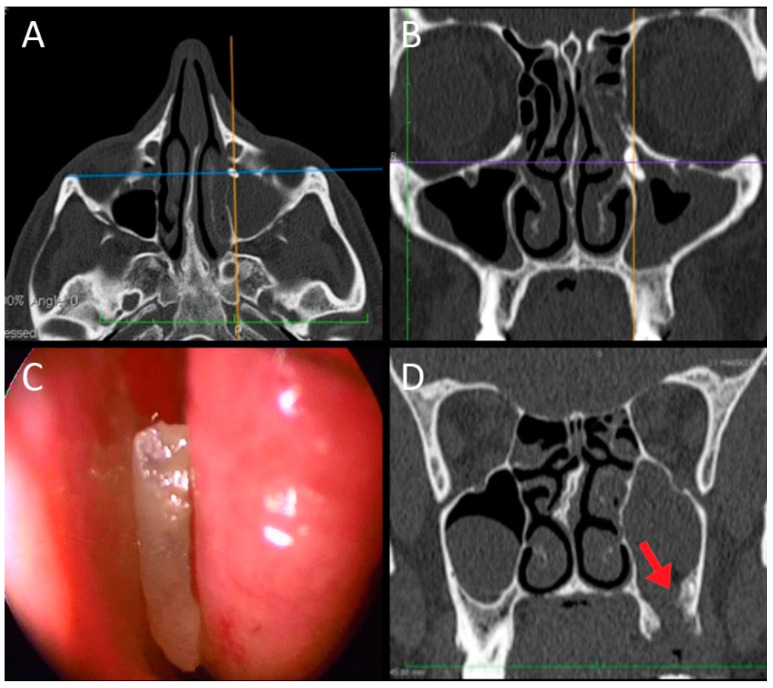
Male patient, 61 years old, complaining of sinus pain, nasal obstruction, and rhinorrhea. CT ((**A**): axial scan; (**B**): coronal scan) revealed the presence of a foreign body in the left osteomeatal complex. Endoscopic surgery was performed to remove the foreign body (**C**) and to clean the maxillary sinus. Because of wide oroantral fistula (**D**), red arrow surgical closure using a local mucosal flap was required.

In Figure 5, a maxillary complication due to a fragment of a root displaced after dental extraction.

Therapeutic modalities included transnasal endoscopic sinus surgery and specific dental management. In 152/262 patients (58%), endoscopic sinonasal surgery and odontoiatric treatments were performed simultaneously during the same surgical procedure and patients were followed closely. In 110/262 patients (42%), odontoiatric treatments were performed in a two-step process based on the type of treatment and specific needs of patients. The overall treatment success rate (self-reported symptom resolution, significant improvement of SNOT-22 and endoscopically observed involved sinus healing) was 96.5% at one year of follow up. In successful patients, we observed significant improvement of the quality of life at 15 days after surgery. In fact, the mean post-operative SNOT-22 was significantly lower compared to baseline: 6 versus 43.4 (*p* < 0.05) at 15 days after surgery. We observed an important improvement in the domains related to physical symptoms, in particular nasal discharge (“thick nasal discharge” and “post nasal discharge”), “facial pain/pressure” and “need to blow nose”. No intraoperative complications (such as bleeding, lamina papyracea damage or cerebrospinal fluid leakage) were reported during endoscopic sinus surgery. No anesthesia-related adverse events were reported. One day of hospitalization was required in 94.8% of patients, a two days in 5.2% of cases. The rate of oroantral fistula (OAF) repair was 95.8% (69/72 patients) and in 3/72 patients (4.2%) a second procedure was required to resolve the problem.

**Figure 6:**
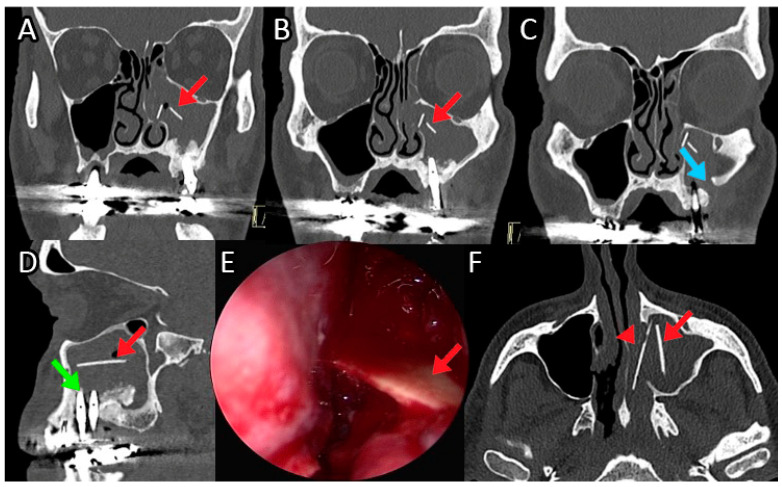
Male patient, 59 years old, complaining of sinus pain, nasal obstruction, and rhinorrhea. CT revealed the presence of a foreign body (2 bars of hydroxyapatite used for the sinus lift preimplantation in the left maxillary sinusitis), as well as maxillary sinusitis, oroantral fistula, and implant displacement with bony reabsorption. Endoscopic surgery was performed to remove the foreign body (**E**) and to clean the maxillary sinus and OAF closure. Persistence of oroantral fistula was observed and was approached by surgical closure using a local mucosal flap. Red arrow in (**A**,**B**,**E**) indicates the foreign body; Light blue arrow in (**C**) indicates OAF; green arrow in (**D**) indicates the implant displacement, and red arrow the foreign body; in (**F**), red arrow and arrowhead indicate the two bars in the axial scan.

Considering data of patients with a poor outcome, we observed that 4 patients required further long-term antibiotic therapy, 3 cases required a second surgical procedure after antrostomy closure and revision surgery for persistent oroantral fistula, and 2 cases required revision surgery for frontal sinus restenosis. Figure 6 shows a complex case of a patient with maxillary sinusitis as a complication of sinus lift and implantation.

## 4. Discussion

Despite its relatively high prevalence, SCDDT has received minimal attention in literature [21,22] decreasing attention and recognition by physicians. Most of the cases of monolateral maxillary sinusitis observed in routine ENT clinical practice are the result of an underlying dental pathology or a consequence of dental surgery. Data of the literature are heterogenoeus. Some studies reported that an odontogenic etiology accounts for 10–30% of cases of maxillary rhinosinusitis, with the percentage increasing to 75% considering symptomatic patients undergoing surgical treatment [23,24,25]. Fredriksson et al. [26] observed in a retrospective study that 40% of cases of radiologically-verified unilateral maxillary sinusitis had an odontogenic origin. Interestingly, Ly et al. [27], observed that SCDDT was found in 48% of cases of unilateral maxillary sinusitis, but one-third of the patients did not undergo dental evaluation. Herein, we report our experience in the management of SCDDT, describing the clinical characteristics, etiology, and outcomes of treatment.

Our data confirm that endoscopic approach represents a less traumatic modality to treat SCDDT, compared to Caldwell-Luc and other osteoplastic approach to maxillary sinus and several studies have demonstrated that it has a high rate of success [17,28,29]. Classic Caldwell-Luc surgery with removal of the innocent anterior wall and of all sinus mucosa and creation of an infraturbinate window has been used, although its disadvantages have been discussed elsewhere in the relevant literature [30,31]. Osteoplastic surgery of the maxillary sinus allows good visualization of entire sinus but it could be complicated with postoperative dislocation of bony fragment into sinus or inflammatory resorption or fracture [32]. Based on our experience, we always prefer an endonasal endoscopic conservative approach not only for sinus toilette and for removal of the odontogenic foreign bodies dislocated in the maxillary sinus, but also for restoration of ostium patency. In this way, the natural sinus clearance mechanism is restored and facilitates faster healing even of OAF is present.

In this series, we present outcomes after endoscopic sinus surgery, reporting an overall treatment success rate of 95.8% in patients undergoing oroantral fistula (OAF) repair. We also demonstrat fast and significant improvement of the quality-of-life of patients as documented by the significantly lower SNOT-22 score at 15 days after surgery compared to baseline. Our results confirm literature data suggesting that patients with significant sinonasal symptoms can benefit from primary endoscopic sinus surgery, which leads to faster symptomatic resolution and subsequently postponed dental treatment. Craig et al. [33] in a prospective cohort study reported that primary endoscopic sinus surgery resulted in significantly faster resolution of sinonasal symptoms, SNOT-22 scores, and endoscopy findings compared to primary dental treatment. More specifically, these variables resolved in 7 to 12 days, whereas patients successfully managed by dental treatment alone resolved in 35 to 56 days.

In our department, patients with SCDDT are managed with a multidisciplinary approach by working in a close collaboration with a dentist and radiologist in all phases of the diagnostic and therapeutic work-up. In agreement with literature data, we believe that close cooperation between different specialists in the management path ensure the greatest opportunity for success and achieve rapid recovery and minimize the risk of recurrence [23,34,35,36,37]. Concomitant treatments should ensure complete resolution of the infection and prevention of recurrence and complications. Fadda et al. [28] argued that gold standards of management require a multidisciplinary approach between an implantologist and maxillofacial/oral and ENT specialists. Our multidisciplinary approach is based on cooperation before surgery in order to achieve correct diagnosis, to discuss the timing and types of interventions and, after surgery, to closely follow healing of the maxillary sinus and resolution of the odontoiatric cause. When possible, we prefer to combine endoscopic endonasal conservative surgery and an odontoiatric approach in a single procedure; if this is not possible, odontoiatric treatment is delayed after surgery. We always discuss and inform patients during pretreatment planning in order to personalize the approach and share the timing and modality of dental/ENT procedures.

Another interesting question is the extent of the endoscopic sinonasal approach, which has not been studied in depth. Some authors have suggested surgical treatment of all sinuses involved by infection, whereas others proposed that an endoscopic approach should be limited to the maxillary antrostomy alone [38,39]. Multiple studies have demonstrated high success rates after opening all diseased sinuses, although comparisons with other studies is difficult because extra maxillary extension of the disease is not always reported. Ungar et al. [40] reported the outcomes of maxillary antrostomy alone for 25 patients with odontogenic maxillary sinusitis, reporting good results in all patients after 3 months. A prospective series by Craig et al. [33] reported that the opening of all diseased sinuses in 26 patients undergoing endoscopic sinus surgery resolved the disease in all cases within 7 to 12 days postoperatively. Accordingly, in our series we always treated all involved sinuses in order to achieve resolution of infection and symptoms as soon as possible.

Another important topic is treatment of the frontal sinus. Some authors believe that frontal sinusotomy is not only unnecessary, but that it is even contraindicated for the high risk of frontal recess mucosal damage and postoperative fibrosis and stenosis. Ungar et al. [40] presented the surgical outcomes of patients who presented with odontogenic maxillary sinusitis involving the frontal sinus and managed by middle meatal antrostomy alone. They concluded that frontal sinusotomy is apparently not necessary to resolve sinusitis. In our series, we always associated frontal sinusotomy especially if pre-existing sino-nasal conditions precluded spontaneous resolution of fronto-ethmoidal sinusitis. We believe that this topic should be further investigated in future studies.

## 5. Conclusions

In our series, iatrogenic causes (55.34%) of maxillary complications were more prevalent compared to dental pathologies (44.65%) according to literature data, showing an increase in maxillary complications related to implant procedures. For this reason we believe nasal surgeon should always be aware that maxillary sinusitis may have not only a rhinogenic cause, but also a dental cause; on the other hand, the dentist/oral surgeon, when treating dental/oral infections, must be aware of the risks of maxillary complications. Our study shows that endoscopic sinus surgery can be a successful surgical procedure for treatment of SCDDT with a success rate of 96.5%, fast resolution of sinonasal symptoms, and improvement in quality of life. In fact, the technique allows resolution of the infection, restoration of proper sinus ventilation, prevention of recurrence, and removal of migrated odontoiatric material or foreign bodies even in challenging cases.

## Figures and Tables

**Table 1:** Demographic and clinical data.

Variable	
Age (years)	50.84 (21–78)
Gender (%) Female	
	59.55% (156/262)
SNOT-22 (mean baseline)	43.4
Symptoms (%)	
Nasal obstruction	61.4% (161/262)
Anterior discharge	86.2% (226/262)
Posterior discharge	74.8% (196/262)
Facial pressure	50.8% (133/262)
Hyposmia	21.7% (57/262)
Cacosmia	43.5% (114/262)
Extension (%)	
Maxillary	38.5% (101/262)
Maxillary + Ethmoid	50.4% (132/262)
Maxillary + Ethmoid + Sphenoid	1.1% (3/262)
Maxillary + Ethmoid + Frontal	9.9% (26/262)
Endoscopic findings (%):	
Edema (hyperplastic mucosa)	49.2% (129/262)
Polyps	18.3% (48/262)
Purulent exudate	85.5% (224/262)

**Table 2:** Distribution of patients in groups and classes.

Groups	Classes	Total (n—%)
I-Preimplantological treatment complications	1.Sinusitis following preimplantologic surgery	22/262 (8.4%)
II-Implantological treatment complications	2a. Sinusitis with peri-implantitis/ subperiostal implant (±OAF *)	36/262 (13.8%)
	2b. Sinusitis following implant dislocation with OAF	6/262 (2.3%)
	2c. Sinusitis following implant dislocation	15/262 (5.7%)
	2d. Implant dislocation	5/262 (1.9%)
III-Classic dental disease or treatment complications	3a. Odontogenic sinusitis with OAF	57/262 (21.7%)
	3b. Odontogenic sinusitis	121/262 (46.2%)

* OAF: oroantral fistula.

## Data Availability

Not applicable.
